# Understanding the patient’s experience of coeliac disease diagnosis: a qualitative interview study

**DOI:** 10.3399/BJGP.2023.0299

**Published:** 2024-01-09

**Authors:** Alice M Harper, Jessica Watson, Rachel O’Donnell, Martha MC Elwenspoek, Jonathan Banks

**Affiliations:** Centre for Academic Primary Care, Population Health Sciences, Bristol Medical School, University of Bristol, Bristol.; Centre for Academic Primary Care, Population Health Sciences, Bristol Medical School, University of Bristol, Bristol.; NIHR Applied Research Collaboration West, Population Health Sciences, Bristol Medical School, Bristol.; NIHR Applied Research Collaboration West, Population Health Sciences, Bristol Medical School, Bristol.; NIHR Applied Research Collaboration West, Population Health Sciences, Bristol Medical School, Bristol.

**Keywords:** coeliac disease, diagnosis, gastroenterology, patient perspectives, primary care, qualitative research

## Abstract

**Background:**

Coeliac disease (CD) presents with non-specific symptoms, and delays to diagnosis are common. The traditional diagnostic pathway involves serological testing followed by endoscopic biopsy; however, the evidence is increasing about the effectiveness of a diagnosis without the need for a biopsy.

**Aim:**

To understand the patient’s experience of being diagnosed with CD.

**Design and setting:**

A qualitative study was conducted, which involved semi-structured interviews with adults diagnosed with CD living in the UK.

**Method:**

Participants (*n* = 20) were purposefully sampled from 200 adults who had completed a diagnostic confidence survey. Interviews were conducted via video-conferencing software (Zoom), recorded, and transcribed verbatim. Data were analysed using reflexive thematic analysis.

**Results:**

Interviewees faced pre-diagnostic uncertainty, presenting with non-specific symptoms that many experienced for several years and may have normalised. GPs often attributed their symptoms to alternative diagnoses, commonly, irritable bowel syndrome or anaemia. Investigations caused further uncertainty, with half of the interviewees unaware that their initial serology included a test for CD, and reporting long waits for endoscopy and challenges managing their diet around the procedure. Their uncertainty reduced once they received their biopsy results. Endoscopy was presented as the ‘gold standard’ for diagnosis and most interviewees believed that the procedure was necessary for diagnostic confidence and conviction in a lifelong gluten-free diet.

**Conclusion:**

Patients experience uncertainty on the pathway to a diagnosis of CD. GPs could improve their experiences by being mindful of the possibility of CD and sharing information about serological testing. Policy and guidance should address the time to endoscopy and diet during diagnosis. If diagnosis without biopsy is adopted, then consideration should be given to clinical pathway implementation and communication approaches to reduce patient uncertainty.

## Introduction

Coeliac disease (CD) is *‘a chronic small intestinal immune-mediated enteropathy precipitated by exposure to dietary gluten in genetically predisposed individuals’*.^1^ The prevalence of diagnosed CD has increased over time and continues to rise throughout Europe.^2^ The widely referenced average prevalence of CD in Europe is 1%;^3^ however, only an estimated 30% of individuals with CD in the UK are diagnosed.^4^

Classically, CD presents with features of malabsorption including diarrhoea, steatorrhoea, weight loss, and/or failure to thrive.^5^ More commonly, CD presents with non-specific symptoms including vague gastrointestinal symptoms, anaemia, and fatigue. Given that CD can present at any age with a wide range of symptoms and signs, a recent review article chronicles the disease as a *‘clinical chameleon’*.^6^ For patients, this may result in delays to diagnosis with a significant impact on their quality of life and potential long-term health consequences, including impaired bone health and malignancy.^7^^–^9 Timely identification, diagnosis, and management of CD is therefore key. The only management currently recommended is lifetime adherence to a gluten-free diet, which improves symptom burden but brings about other challenges including impact on quality of life.^10^

Traditionally, a diagnosis of CD is made by serological testing, followed by endoscopic biopsy while consuming a gluten-containing diet.^5^^,^^11^ As a result of progress in serological testing and restricted access to endoscopy services exacerbated by the COVID-19 pandemic, the British Society of Gastroenterology published interim guidance for adults in 2020 that advised that people aged <55 years with symptoms consistent with CD and no alarm symptoms can be diagnosed without endoscopic biopsy if immunoglobulin A tissue transglutaminase is >10 times the upper limit of normal and a further immunoglobulin A endomysial antibodies test is positive.^12^^,^^13^ This reflects increasing evidence about the effectiveness of serological testing without biopsy.^14^^,^^15^

**Table:** How this fits in

Previous qualitative studies on coeliac disease (CD) focus on the patient’s experience after diagnosis. This study found that patients experience uncertainty during the pathway to a CD diagnosis, particularly pre-diagnosis and during investigations. Endoscopy was thought to be necessary for diagnostic confidence and conviction in a lifelong gluten-free diet. As the diagnostic pathway evolves, consideration must be given to reducing patient uncertainty.

Research examining the patient’s experience of being diagnosed with CD is limited. Qualitative studies tend to focus on life after diagnosis and the gluten-free diet.^16^^–^22 A survey of 276 adult members of Coeliac UK published in 2020 highlighted that 70% spoke to their GP before diagnosis, 20% felt that their symptoms were not taken seriously, and 48% reported that time to diagnosis was slow.^23^ Studies in Australia and France explored the diagnostic experiences of patients and found that they experienced a range of symptoms before diagnosis, encountered delays to diagnosis causing uncertainty and/or frustration, and had variable emotional responses to diagnosis, often including initial relief.^24^^,^^25^ Despite the challenges surrounding the diagnosis of CD, no further studies and no UK-based studies have examined the diagnostic experience of patients with CD. This study therefore aimed to identify and understand the patient’s pathway to diagnosis of CD and how it might be improved.

## Method

### Study design

Semi-structured qualitative interviews were carried out with people diagnosed with CD. The study used an interpretive approach, seeking to understand how people make sense of the CD diagnostic pathway and their responses to these experiences.^26^ The research team comprised researchers (clinical and non-clinical) with backgrounds and interests in qualitative methods, primary care, diagnostic testing, and coeliac disease. Two patient representatives, one with a son, husband, and father all diagnosed with CD, and another who had been diagnosed with CD, both contributed to protocol development, patient information and consent form content, and topic guide development, and conducted pilot interviews.

### Participants

Participants were purposefully sampled from 200 adults with a diagnosis of CD who had previously completed a survey on desired levels of diagnostic confidence before commencing a gluten-free diet and their views on the need for a biopsy.^27^ Criteria used for sampling participants were age, sex, and level of diagnostic confidence (low, medium, or high). A total of 62 adults with CD, who had given permission to be contacted as part of the survey, were sampled and invited to participate via emails and a follow-up email sent at 2 weeks. Once an online consent form was completed, an interview was arranged with the first 20 participants who responded, and they received a £20 voucher for their time. Data collection continued until saturation was achieved. Participants provided written informed consent to participate including audio-recording and publication of anonymous quotations, with confidentiality preserved.

### Data collection

One researcher, a female academic GP trainee with previous qualitative interview training, conducted all interviews between November 2021 and February 2022. Interviews were conducted and recorded via telephone and audio-recorder or video- conferencing software (Zoom), according to participant preference. Interviews lasted between 20 and 50 minutes, with a mean duration of 32 minutes.

An interview topic guide (see Supplementary Table S1 for details) was iteratively developed by the research team to collect data on the diagnostic process; factors that triggered CD testing; information communicated by clinicians around investigations; and, if relevant, the impact of COVID-19 on the diagnostic process. Interviews were flexible to allow exploration of topics raised by participants. Data were stored in accordance with the General Data Protection Regulation and the University of Bristol’s data protection policies.

### Data analysis

Interviews were professionally transcribed, anonymised, and uploaded into NVivo (version 12) to support analysis. Data were analysed using reflexive thematic analysis, an iterative process involving data familiarisation, coding, theme generation, theme reviews, theme definition, and report write up.^28^ Three transcripts were used by one researcher to develop a coding framework that was then reviewed and refined by two other researchers. All three independently coded two transcripts, with the original researcher then completing full coding of all transcripts in discussion with the research team who collaboratively developed the key themes.

## Results

### Interviewee characteristics

In total, 20 interviews were conducted. Most interviewees were female (90%) and had been diagnosed with CD within the last 10 years (80%). Interviewee characteristics are presented in Table 1. Although participants were not sampled according to professional/employment characteristics, interview data did highlight that six interviewees held or had held clinical and/or scientific roles.

**Table 1.** Interviewee characteristics (*n* = 20)

**Characteristic**	***n***
**Sex**	
Male	2
Female	18

**Age, years**	
18–25	6
26–40	5
41–64	6
≥65	3

**Age at diagnosis, years**	
≤17	2
18–25	6
26–40	4
41–64	7
≥65	1

**Time since diagnosis, years**	
<5	9
6–10	7
11–15	2
16–19	0
≥20	2

### Pre-diagnostic uncertainty and non-specific presentation

Interviewees frequently presented to their GP with gastrointestinal symptoms or fatigue, which are prevalent in primary care and non-specific. Gastrointestinal symptoms commonly mentioned included abdominal pain and/or distention and constipation and/ or diarrhoea. Most interviewees had experienced symptoms for a long time, sometimes for years, before diagnosis. This resulted in some interviewees normalising their symptoms and continuing with their daily lives until symptoms impacted on their quality of life:

*‘I think I kind of just had all these weird symptoms for about three years before I thought “Right, actually I’m just not … I’m not getting over whatever this is and I need to go to the doctors” … I just kind of thought “This is how I am, this is normal for me” and I just got to a point where I was just so down about not feeling normal and I’d forgotten what normal felt like.”’*

(CD1, female, aged 26–40 years, 5–10 years since diagnosis)

Before receiving a diagnosis of CD, symptoms were often attributed to alternative diseases such as irritable bowel syndrome (IBS) or anaemia by interviewees and GPs. However, for some, these conditions did not align with their symptoms and illness experience:

*‘I’ve basically been backwards and forwards to the doctors, the GPs, for many, many years, probably getting on for close enough twenty years or more, just being told that I had IBS. I was constantly anaemic, could never take iron tablets because they never agreed with me and it was just frustrating my body. I was just told “You’ve got IBS, got to get on with it and watch what you eat” and I did watch* [what] *I eat. I ate very, very carefully, take peppermint capsules, they might help you, and it wasn’t … I just kind of gave up GP appointments and then I think two years after that I thought I can’t cope any more, there was more going on.’*

(CD16, female, aged 41–64 years, <5 years since diagnosis)

Furthermore, interviewees felt, particularly in relation to IBS, that they were assigned a diagnosis that was being used as a convenient ‘catch all’ for patients who did not fit neatly into diagnostic categories. In the example below the participant expresses a rejection of IBS because it is used so ubiquitously and lacks diagnostic certainty:

*‘I mean initially the doctor kind of said IBS and I just thought “Ah, do you know what, I’m not happy with being diagnosed with IBS because I kind of feel like everyone and their granny gets diagnosed with IBS, you know, your dog can have IBS”* [laughs]*.’*

(CD1, female, aged 26–40 years, 5–10 years since diagnosis)

Around half of interviewees felt that CD could have been explored earlier. Those who had symptoms attributed to IBS and/or anaemia felt that these diagnoses had prevented further investigations for CD:

*‘I kind of wish they’d pursued an actual cause or an actual disease when I was younger rather than just sort of slapping the placeholder irritable bowel syndrome on it because I don’t think that’s very helpful to anyone.’*

(CD5, male, aged 26–40 years, 5–10 years since diagnosis)

*‘I was forty-two when I was diagnosed but I had always been anaemic and I know that’s one of the prime symptoms isn’t it? But nobody ever investigated it, it was just “Oh, you’re a woman, you know, whatever.” And that had just gone on for years with no further sort of investigation and no, I hadn’t had any other symptoms at all.’*

(CD3, female, aged 41–64 years, >20 years since diagnosis)

Faced with non-specific presentations, the interviewees’ GPs tended to use more prevalent differential diagnoses to explain the symptoms that patients were experiencing. In some cases, this appeared to prevent alternative diagnoses, including CD, being considered.

### Mid-diagnostic uncertainty and investigations

Participants described their investigations for CD, which involved serological testing in the first instance. This usually started with blood tests performed in primary care and only half of the interview group were aware that these tests would include coeliac serology, with the possibility of the condition not discussed:

*‘I didn’t know what he was testing for, I just knew he was going to be doing some tests … It would be interesting now for me to ask what else I was tested for but the truth is I don’t know.’*

(CD2, male, aged 41–64 years, 5–10 years since diagnosis)

Interviewees often described feelings of surprise on receiving positive coeliac serology results. The surprise element arose as they were unaware that their symptoms may be caused by CD. Indeed, some had no previous awareness of the disease itself. This was usually followed by a positive emotional response as they had an explanation for their symptoms:

*‘The doctor just did a blood test and said oh we’ll just add it into* [laughs] *the other blood tests she was taking, so her and me were both very surprised when it came back positive.’*

(CD14, female, aged 41–64 years, 5–10 years since diagnosis)

*‘Went to the doctor, he did some tests, told me I had tested positive for the coeliac antibodies. That was the very first time I’d even heard of the disease. I had a look on Google at the symptoms, as you do, and literally as I went down the list ticked every single box, not just one here or there, it’s every single one. I thought “Oh my God, yeah.” Basically at that point I was pretty certain what the diagnosis would eventually be. But no, I’d never heard of it beforehand, I didn’t suspect I had it or anything.’*

(CD2, male, aged 41–64 years, 5–10 years since diagnosis)

Triggers for serological testing in those living with symptoms for a long time were recurrent presentations or a consultation with a different GP. The length of time taken to investigate for CD was a source of frustration because interviewees felt the possibility could have been explored earlier.

Most interviewees were advised by their GP that they would require endoscopy and biopsy after receiving results of coeliac serology. Endoscopy was presented as the necessary next step on the diagnostic pathway, the ‘gold standard’ investigation for diagnostic confirmation of CD:

*‘She said I would hundred per cent* [need an endoscopy] *because that’s the only way to diagnose it fully.’*

(CD19, female, aged 26–40 years, <5 years since diagnosis)

Most interviewees believed that an endoscopy was necessary to confirm the diagnosis before committing to a lifelong gluten-free diet as well as excluding alternative diagnoses. Others were happy with a serological diagnosis but were advised by their GP to proceed with an endoscopy:

*‘I was trying to chase up the endoscopy, not that I really wanted one. I seriously considered whether I wanted one or not and then he suggested that it was quite a good idea and I said “OK”. So I just kept trying to chase it.’*

(CD10, female, aged 41–64 years, <5 years since diagnosis)

Duration of time from positive serology to endoscopy varied from weeks to months. Long waits were difficult so a number of interviewees sought endoscopy via private health care to reduce the wait time. There was considerable uncertainty for interviewees in managing their diet leading up to an endoscopy. Those who had commenced a gluten-free diet after their serology results faced a challenging predicament because they were required to re-commence a gluten-containing diet for an accurate biopsy result:

*‘I was in this ridiculous situation that I didn’t know when the endoscopy would come through but at the same time when I had the endoscopy I had to be eating gluten again for eight weeks, so then I didn’t know when to start eating gluten, do you see what I mean, ‘cos initially I’d gone off of it straightaway and over a period of time things eased in my tummy but then I knew I had to go back on it again to have this endoscopy done so then I was in this sort of when do I do it because I was on a waiting list.’*

(CD14, female, aged 41–64 years, 5–10 years since diagnosis)

Those who adhered to a gluten-containing diet usually continued to experience symptoms and were distressed while waiting for an endoscopy appointment knowing that their symptoms would improve when they could stop eating gluten:

*‘So basically I was just waiting on an appointment coming through the post and then when it did come through the post I’m sure it was about three months or something I was going to have to wait before I could get the endoscopy, which in my eyes was horrific because I just wanted to stop eating gluten.’*

(CD1, female, aged 26–40 years, 5–10 years since diagnosis)

In summary, during investigations for potential CD, interviewees continued to face uncertainty, be that a lack of awareness that their blood tests included coeliac serology and/or the timing of their endoscopy and management of their diet while awaiting the procedure.

### Diagnostic confirmation and reflection on experience

Once they received endoscopy results, the uncertainty that interviewees had experienced around their symptoms and diagnostic investigations was reduced. In general, interviewees accepted the diagnosis and associated dietary changes. Many used the time spent waiting for an endoscopy to undertake research on the condition and adjust to the possibility of living with CD:

*‘He started telling me all about it but by then I knew all about it, I knew about the villi, is that right? Yeah, I knew that everything would be flat and I wouldn’t have any so he was starting to draw me diagrams and it was, “Yeah, I know it now, I’ve researched everything now.”’*

(CD16, female, aged 41–64 years, <5 years since diagnosis)

Reflecting on confidence in their diagnosis of CD, most interviewees felt that endoscopy results offered the definitive diagnosis. Those who considered that endoscopy may not have been necessary to confirm their diagnosis still believed that the procedure offered certainty:

*‘I didn’t feel I needed that but yeah, I understood the need for … I understood the need for it as a rubber stamp.’*

(CD2, male, aged 41–64 years, 5–10 years since diagnosis)

Having an endoscopy offered most interviewees conviction in following a lifelong gluten-free diet, increasing adherence with the diet for some, as well as reducing any post-diagnostic uncertainty if symptoms recurred:

*‘I think it was reassuring to have, then incontrovertible evidence and, I guess, I thought that the endoscopy was maybe like clearer evidence or that had been kind of portrayed to me like that, but definitely that, yeah, the combination of those two was, yeah, I felt then satisfied and that there was actually no doubt, which I think was important because I continued to kind of have stomach pains a little bit continually and it was reassuring to know that it definitely was that and that I just needed to work a bit harder on the diet rather than be potentially worrying that it could be something else as well.’*

(CD7, female, aged 18–25 years, 5–10 years since diagnosis)

*‘Because I’ve had like the proper official diagnosis I feel like I take the diet a lot more seriously than perhaps I would have before, like if I hadn’t have had that test.’*

(CD11, female, aged 18–25 years, <5 years since diagnosis)

One interviewee was diagnosed from positive coeliac serology alone during the COVID-19 pandemic. She was initially told she should proceed with an endoscopy, despite not being keen, and was then passive towards a change in her diagnostic pathway:

*‘I was quite happy about it* [laughs]*, I wasn’t bothered. The only thing I would have been interested in knowing was the state of my intestines really … I just go with the flow really. If they’re telling me I don’t need one I don’t need one, I’m not going to push for one.’*

(CD10, female, aged 41–64 years, <5 years since diagnosis)

When considering accepting a diagnosis from serology alone, other interviewees wanted to ensure ‘certainty’ or ‘conclusivity’:

*‘I don’t really know anything about the blood results to be honest, I don’t know how sure you can be from the blood results but because they said it’s not a certainty I would certainly have wanted to make sure I knew for sure.’*

(CD12, female, aged 26–40 years, 10–15 years since diagnosis)

Finally, when reflecting on seeking a diagnosis of CD, interviewees flagged the importance of increasing awareness of the symptoms of CD among the general population for earlier presentation, as well as among GPs and allied healthcare professionals for earlier testing:

*‘I have a fairly distant friend who’s got coeliac disease and I wish I’d paid more attention to that and noticed what that was.’*

(CD2, male, aged 41–64 years, 5–10 years since diagnosis)

*‘I suppose it would have been nice to know this before so that, yeah, I didn’t feel so rubbish for so long.’*

(CD8, female, aged 26–40 years, <5 years since diagnosis)

## Discussion

### Summary

In this novel qualitative study, patients described experiencing uncertainty during the diagnostic process for CD. Before diagnosis, interviewees presented with non-specific symptoms. Such symptoms were sometimes present for several years and may have been normalised by interviewees or attributed to an alternative diagnosis, such as IBS or anaemia by GPs. During investigations, half of interviewees were unaware that their serology included a test for CD. Many encountered long waits for NHS endoscopy services and further uncertainty managing their diet around the procedure. Their uncertainty was reduced once they received biopsy results. Endoscopy was presented, usually by GPs, as the ‘gold standard’ investigation for diagnosis and most interviewees believed that endoscopy was necessary for diagnostic confidence and conviction in a lifelong gluten-free diet.

### Strengths and limitations

The main strength of this study was the use of qualitative methods for a detailed exploration of interviewees’ views and experiences of their path to CD diagnosis. The study benefited from a previous survey in which interviewees were purposefully sampled, including those with different stances on individual diagnostic confidence.^27^ Sample size was appropriate and data saturation achieved.

The study was limited predominantly to females diagnosed within the previous 10 years, and data on ethnicity were not collected. Findings may not therefore be representative of males or particular ethnic groups, although a greater proportion of females than males have CD and females are at higher risk of CD than males. Western ethnicity is also associated with a 6.85 higher odds of CD autoimmunity.^29^^–^31 Interviewees self-selected to participate in the research and six people reported being from a clinical or scientific background, potentially introducing bias to the sample. Data were collected from interviewees’ recollection of their experiences, which may introduce recall bias. Given that half of the participants had been diagnosed >5 years ago, their experiences may not reflect recent increased awareness of CD in primary care and/or development of diagnostic pathways. Patients’ experiences during endoscopy were not explored, which is an important aspect of the diagnostic process.

### Comparison with existing literature

Delays in the diagnosis of CD are widely reported.^8^^,^^14^ One UK population survey study reported a mean duration of symptoms of 13.2 years before CD diagnosis.^9^ The current study adds to existing qualitative data exploring patient experiences before diagnostic tests. Reporting on the many patients who experience symptoms for years before diagnosis, Houbre *et al*^25^ described how *‘the subjects and physicians therefore attempted to find different explanations’*. Similar to the findings of Taylor *et al*,^24^ the current study found that some interviewees normalised their symptoms and were unaware they may be indicative of disease; indeed, some interviewees had no, or very limited, previous awareness of CD.

Systematic review findings suggest that anchoring (supporting the original diagnosis at review) and confirmation bias (prioritising information that supports an original diagnosis) are associated with diagnostic and management errors in medicine.^32^ The current study found evidence of these aspects in participants’ accounts, in particular, the different explanations of symptoms that participants received from healthcare professionals, with a number reporting diagnoses of anaemia or IBS. CD was only considered after multiple presentations or after consulting a different doctor. These experiences point toward GPs holding cognitive biases, including anchoring and confirmation bias, which can block accurate diagnosis. Improved awareness of CD and associated symptoms could therefore reduce the time to diagnosis and improve the patient experience. Both patients and GPs need to be alert to the *‘clinical chameleon’* of CD.^6^

Recent research on investigative and testing decisions in primary care has highlighted a lack of information sharing and shared decision making.^33^^,^^34^ The findings from the current study are also indicative of this tendency in relation to CD; interviewees reported that, while coeliac antibodies were included as part of multiple serological tests, only half were aware they were being tested for CD.

There are challenges for patients who may have CD in managing the time between positive serology results and endoscopy. Survey data collected from Coeliac UK members have highlighted long wait times for endoscopy.^23^ Interviewees in the current study identified the uncertainty they experience about their diet while awaiting endoscopy, particularly for those who commence a gluten-free diet after receiving positive serology results. This study also contributes a novel understanding of patients’ perceptions about the need for endoscopy during the diagnostic process of CD. With increasing evidence and support for ‘biopsy-free’ diagnostic strategies, consideration should be given to instilling confidence in patients about the diagnosis and subsequent commitment to a lifelong gluten-free diet.^12^^,^^14^^,^^15^

A 2021 overview of the literature on patient and clinician experiences of uncertainty during diagnostic processes describes steps where uncertainty occurs, the aetiology of each area of uncertainty, and strategies to manage uncertainty.^35^ Many of the drivers of uncertainty for patients summarised in this overview complement the experiences of the interviewees in the current study, for example, patients’ uncertainty about the meaning of symptoms, when to seek help, the meaning of tests, and how clinicians interpret and combine diagnostic information.^35^ Recommended management strategies involve acknowledgement of uncertainty and improved communication.^35^ Although a 2021 review on the communication of diagnostic uncertainty in primary care identified significant evidence gaps, general recommendations have been published in the wider literature, and novel tools are in development, such as personalised patient summary leaflets to aid communication around diagnostic uncertainty in primary care.^36^^–^38

### Implications for research and practice

This study highlights the pre-diagnostic uncertainty faced by patients, largely because of the non-specific symptoms associated with CD. GPs should therefore be mindful of the possibility of CD, particularly for those patients diagnosed with IBS or anaemia who re-present with ongoing symptoms. Awareness of potential cognitive biases and correction strategies, such as guided reflection and conscious consideration of alternative diagnoses, may reduce delays to diagnosis.^39^

The study also found that patients face uncertainty during the mid-diagnostic or investigative phase. After considering the possibility of CD, sharing information about serological testing could improve patients’ experiences. With ongoing restricted access to endoscopy services resulting from NHS pressures, it is vital that patients receive information about diet and anticipated endoscopy wait times. Giving at least 3 months’ notice before endoscopy appointments would allow patients to ensure that gluten was included in their diet for sufficient time for biopsy diagnosis where relevant.^40^ Future guidance should address diet during the diagnostic process.

Adoption of serological diagnostic strategies may reduce patient uncertainty during the mid-diagnostic/investigative phase and free up endoscopy capacity. Future research should address whether more patients can benefit from a diagnosis without the need for biopsy and how this pathway should best be implemented. New clinical pathways, including a no biopsy approach, should ensure that patients receive adequate follow up and specialist support to reduce the risk of misdiagnosis. Endoscopy might therefore no longer be presented as the ‘gold standard’ and healthcare professionals would have to develop communication approaches to empower patients with knowledge about the accuracy of serological tests and subsequent confidence in their diagnosis and commitment to a lifelong gluten-free diet.
